# The Influence of Cellulose-Type Formulants on Anti-*Candida* Activity of the Tyrocidines

**DOI:** 10.3390/antibiotics10050597

**Published:** 2021-05-18

**Authors:** Yasamin Masoudi, Wilma van Rensburg, Bernice Barnard-Jenkins, Marina Rautenbach

**Affiliations:** BIOPEP^TM^ Peptide Group, Department Biochemistry, Stellenbosch University, Private Bag X1, Matieland, Stellenbosch 7602, South Africa; yasamin@sun.ac.za (Y.M.); bbarnard@sun.ac.za (B.B.-J.)

**Keywords:** antimicrobial peptide, cyclic peptide, tyrocidine, *Candida albicans*, formulation, cellulose

## Abstract

*Candida* species are highly adaptable to environmental changes with their phenotypic flexibility allowing for the evasion of most host defence mechanisms. Moreover, increasing resistance of human pathogenic *Candida* strains has been reported against all four classes of available antifungal drugs, which highlights the need for combinational therapies. Tyrocidines are cyclic antimicrobial peptides that have shown synergistic activity with antifungal drugs such as caspofungin and amphotericin B. However, these cyclodecapeptides have haemolytic activity and cytotoxicity, but they have been used for decades in the clinic for topical applications. The tyrocidines tend to form higher-order structures in aqueous solutions and excessive aggregation can result in variable or diminished activity. Previous studies have shown that the tyrocidines prefer ordered association to celluloses. Therefore, a formulation with soluble cellulose was used to control the oligomer stability and size, thereby increasing the activity against *Candida* spp. Of the formulants tested, it was found that commercial hydroxy-propyl-methyl cellulose, E10M, yielded the best results with increased stability, increased anti-*Candida* activity, and improved selectivity. This formulation holds promise in topical applications against *Candida* spp. infections.

## 1. Introduction

Out of 350 heterogeneous *Candida* species, more than 17 different *Candida* species are responsible for human and animal infections. However, the majority of invasive infections are caused by *Candida albicans*, *C. glabrata*, *C. parapsilosis*, *C. tropicalis* and *C. krusei* [1,2]. *Candida spp.* can quickly adapt to new and challenging environments in nature. They can use different nutritional resources and express different metabolic pathways for optimal survival. This phenotypic flexibility gives *Candida spp.* the ability to survive various host-defence mechanisms [3,4]. The recent emergence of fungi that are resistant to more than one class of antifungal drug is a serious concern. Currently, polyenes (i.e., amphotericin B), azoles (i.e., fluconazole) and echinocandins (i.e., caspofungin) are the main antifungal drug classes used to treat fungal infections [5]. Therefore, if an antifungal drug class becomes ineffective in treating a fungal infection, it reduces the therapeutic options by at least 33%, and in the most severe resistance cases, clinicians are left with no treatment options.

Unlike resistance in bacteria, there are no known transposon- and plasmid-like resistance mechanisms for antifungal drug resistance [5]. This resistance appears to be multifactorial, involving both mechanisms similar to planktonic antifungal resistance and mechanisms specific to the biofilm lifestyle [6,7]. *Candida* species can alter the concentration and/or structure of antifungal target proteins, as well as transcription factors or enzymes through genetic mutations of their genes [5]. Furthermore, *Candida* species use efflux pumps to reduce the drug concentration, as well as being able to change the sterol composition of the cell membrane [5,8]. With the ever-increasing number of immune-suppressed individuals and increasing *Candida* biofilm research, resistance is a problem that will lead to increased fatalities. Novel antifungals such as antifungal peptides (AFPs) and their synergistic combinations with current drugs could offer a viable option to combat resistant fungal infections. There are a number AFPs with potent antifungal activity that show potential for combination treatments such as plant and insect defensins [9], human defensins [10] and peptides produced by soil bacteria [11,12]. Furthermore, antifungal peptides generally have activity both towards membranes and intracellular targets making the development of resistance less likely [13,14].

Antimicrobial peptides with potential as antifungals are the tyrocidines (Trcs) and analogous tryptocidines (Tpcs). They are small, cyclic decapeptides produced intracellularly [15] by the Gram-positive soil bacterium, *Brevibacillus parabrevis,* in the late logarithmic growth phase as part of a peptide complex, tyrothricin [16]. They have a broad spectrum of activity against Gram-positive bacteria [17,18,19], filamentous fungi [12,20,21] and *Candida albicans* [11], as well as low nanomolar activity against the human malaria parasite *Plasmodium falciparum* [22,23]. Furthermore, Trcs have been found to have synergistic activity against *C. albicans* biofilms when tested in combination with the known antifungal drugs, caspofungin and amphotericin B [11]. However, they have haemolytic and leukocytolytic properties which has limited their clinical use to only as topical applications [24,25].

**Figure 1:**
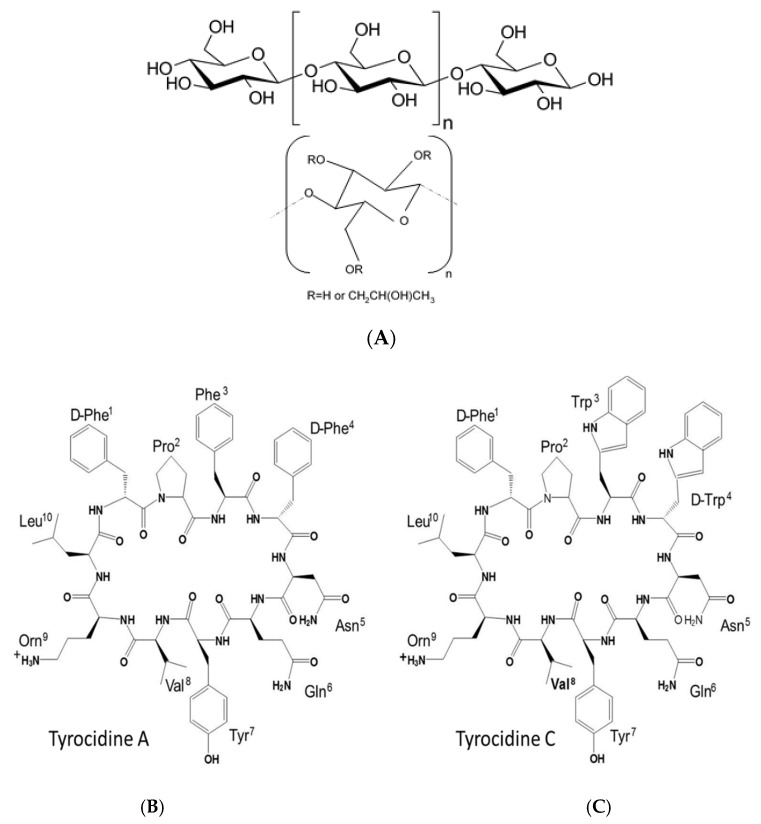
Examples of cellulose derivatives and Trcs studied with (**A**) cellulose polymer, (**B**) core of hydroxyl and methylcellulose derivatives and (**C**) primary structures of tyrocidine A and C.

Trcs refer to a mixture of peptide analogues with a general structure of *cyclo*-(f^1^P^2^X^3^x^4^N^5^Q^6^X^7^V^8^O^9^L^10^) with variances occurring at position 3 and 4 (X^3^x^4^ = Phe^3,4^, Trp^3,4^), position 7 (X^7^ = Tyr^7^/Phe^7^/Trp^7^) and position 9 (O^9^ = Orn^9^/Lys^9^) (D-enantiomer of the amino acid residues are indicated in lowercase) (Figure 1C) [18,26,27]. In an aqueous solution, the cyclic peptide has an antiparallel β-sheet secondary structure that is stabilised by four intramolecular hydrogen bonds [28,29,30,31,32,33], which, in combination, gives rise to their solvent and temperature stability [34,35], but also forms the base for the amphipathic character of the Trcs [28]. Trcs exhibit their amphipathic character only as b-sheet dimers [32,33], which have been proposed to be the active conformation correlated to their membranolytic antimicrobial activity [31]. Furthermore, the range of activity of the Trcs is highly dependent on their oligomerisation profile [36,37,38], as the Trcs readily form aggregates or higher-order oligomers [33,34,36,39], including highly ordered nanostructures [40]. This oligomerisation is dynamic and depends on the solvent environment, cyclodecapeptide identity and feedstock concentration [33,34,36,39,40].

Modification of a peptide’s amphipathicity, hydrophobicity and conformational flexibility in solution, either via chemical modification or conformational changes due to specific interactions with media components or oligomerisation, can influence the peptide’s activity [32,41,42]. It is therefore hypothesised that the formulation of tyrocidines could alter their conformation, oligomerisation, and subsequently, their biological activity [36].

Trcs have been shown to readily associate to various materials, with a preference for cellulose, while maintaining their activity against Gram-positive bacteria [43,44]. The observed association resulted in oligomerisation on the cellulose surface that depended on the peptide solvent system, as well as the absolute peptide concentration [45]. Juhl et al. [46] showed that three of the Trcs, TrcA, B and C, interact specifically with glucose and cellotetraose. They observed that glucose influences the interpeptide hydrogen network, and therefore, oligomerisation. It can therefore be hypothesised that soluble cellulose derivates could affect peptide oligomerisation and possibly modulate the formation of large Trc oligomers [33,47,48]. If the cellulose-type formulation stabilises oligomers such as the amphipathic dimers in solution, it could enhance the overall biological activity. Formation of big aggregates/oligomers would result in the loss of peptide activity due to the loss of peptides from the solution, and possibly the loss of dimeric peptides that are proposed to be the active structures [32,33]. The cellulose derivatives chosen as Trc formulants have a cellulose backbone with different modifications including, methyl, propyl and hydroxyl groups (Table 1).

This study aimed to find the best commercially available cellulose derivative as a formulant for the tyrocidine mixture (Trc mix) from the classic antibiotic complex tyrothricin. The specific focus was to obtain the highest activity and specificity against two *Candida* strains, as well as to stabilise the cyclodecapeptides in solution. To achieve this aim, anti-*Candida* activity assays were performed on the various Trc mix formulations with selected commercial derivatives of cellulose (Table 1). Furthermore, fluorescence assays were performed on the most active formulants of the Trc mix to investigate the effect of cellulose derivatives and maturation time on the oligomerisation behaviour of the peptides in the Trc mix. Lastly, the effect of the formulants on the Trcs’ toxicity was determined against erythrocytes.

## 2. Results and Discussion

The cyclodecapeptides in the Trc mix have a high propensity to oligomerise [33,34,36,39]. Various noncovalent and hydrophobic interactions may be important in the formation of higher/larger oligomers. The presence of four aromatic amino acid residues and three hydrophobic residues in tyrocidines (refer to Figure 1C) could drive hydrophobic interactions in aqueous systems between the peptides to form oligomers. This oligomerisation leads to the change in their optical character, such as far-UV circular dichroism [40,46] and fluorescence emission [40,43], as well as possibly changing their biological activity.

**Figure 2:**
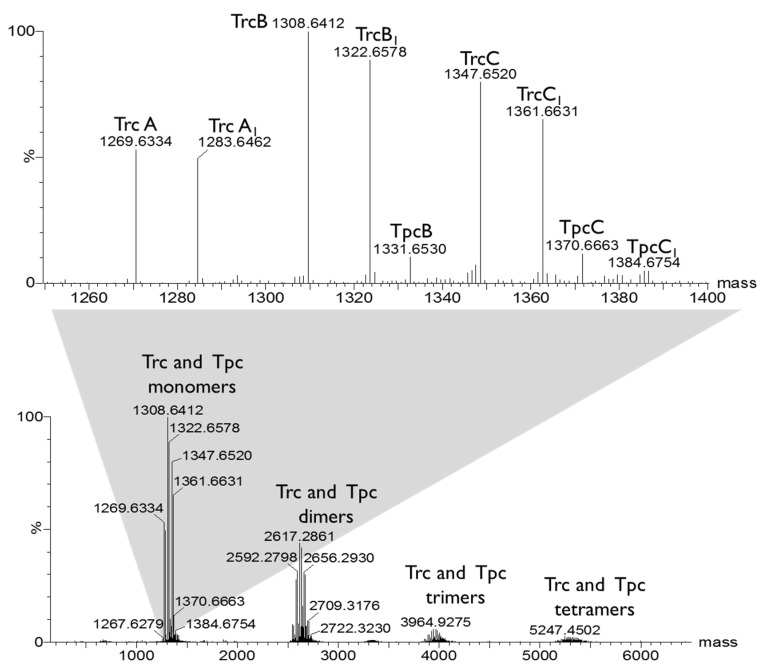
ESMS analysis of the cyclodecapeptides in the Trc mix. The mass spectra were generated with the MaxEnt 3 algorithm in MassLynx 4.01. The top spectrum shows the monomers in the Trc mix and the bottom spectrum shows the homo- and hetero-oligomers that were detected.

A recent study showed that tryptocidine C, a Trp-rich analogue found in the Trc mix, formed defined nanoparticles and that the oligomerisation can be followed by electrospray mass spectrometry and fluorescence [40]. In this study, we observed the oligomerisation of the peptides to form dimers, trimers and tetramers via electrospray mass spectrometry, as depicted in Figure 2. We also followed this oligomerisation with fluorescence which is discussed later in detail. The oligomerisation of the Trcs can lead to instability in the solution and, in turn, this can lead to changes in its activity profile. To modulate the oligomerisation, we prepared six different Trc mix formulations with commercial cellulose derivatives (1:1, *m*/*m*) (Figure 1, Table 1), and compared them with the control Trc mix in 1.5% ethanol in water (*v*/*v*).

The target organism in this study was an environmental strain of *C. albicans* CAB1653 that was isolated from mosquito larvae. We found that this strain was completely resistant towards fluconazole, while it exhibited MICs of 9 mM and 23 mM against caspofungin and amphotericin B, respectively, after a 24 h challenge (refer to Table S2 in Supplementary data). The loss of one of the drug groups and the rather high MIC towards amphotericin B is alarming, as it means mosquitos could be vectors that can spread this resistant *Candida* strain [49]. The cyclodecapeptide complex in the Trc mix exhibited similar or better anti-*Candida* activity than caspofungin and amphotericin B against the planktonic cultures (Table S2). This makes the cyclodecapeptide complex in the Trc mix a viable option to further formulate and investigate as an antifungal agent against *C. albicans* CAB1653.

**Figure 3:**
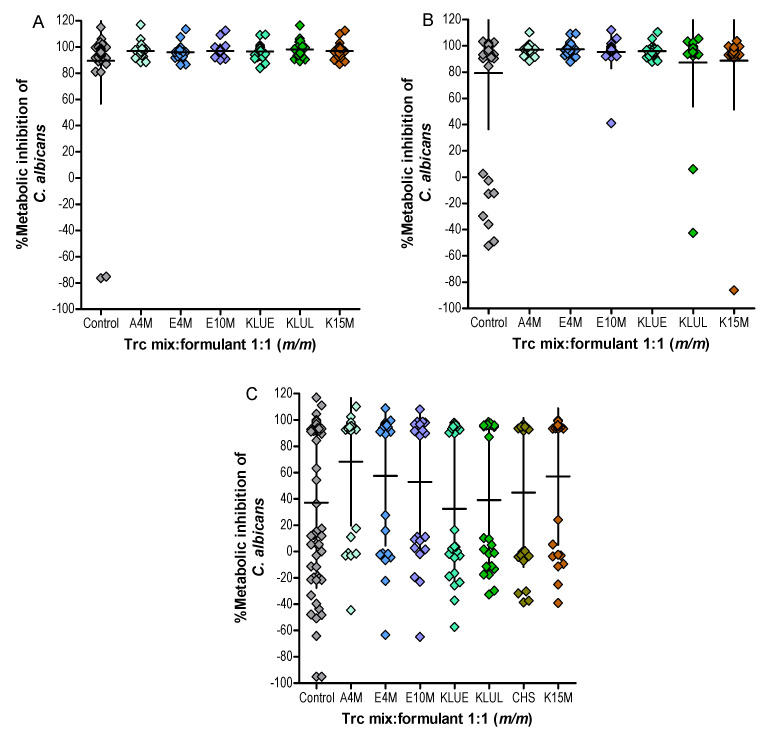
Metabolic inhibition of *C. albicans* cultures treated by formulated Trc mix at 50 µg/mL (**A**), 25 µg/mL (**B**) and 12.5 µg/mL (**C**). Each data point represents one treated culture. The mean % metabolic inhibition, calculated in terms of growth controls, is shown for each condition with standard deviation (SD).

For the six formulations of the Trc mix, the anti-*Candida* activity was measured after 24 h at three concentrations 50.0 µg/mL, 25.0 µg/mL and 12.5 µg/mL to assess the variability in activity and facilitate the selection of the best formulant to stabilise the Trc mix oligomers (Figure 3). The lack of conversion of the metabolic dye resazurin to resorufin was equated to cell death, while survival was equated to the conversion in this assay. The culture survival data derived from Figure 3 are summarised in Table 2. The six formulations with the Trc mix at 50.0 µg/mL exhibited a 100% inhibition of metabolism (0% survival) of all the exposed cultures whereas, for the control Trc mix, 2/55 cultures survived the treatment (4% survival) (Figure 3A). In the 25.0 µg/mL Trc mix, all the formulations were more active than the control with 0–8% culture survival. The 25.0 µg/mL control Trc mix led to a 16% survival, and the conversion of the metabolic dye by the surviving cultures showed a large variation including “negative” inhibition values (Figure 3B). This negative result is due to a higher dye conversion in these cultures, which indicates a metabolic stress response.

Between 29% and 58% of the cultures survived the treatments containing 12.5 µg/mL The Trc mix alone and the Trc mix with cellulose formulants (Table 2). However, these surviving cells exhibited an increased conversion of the metabolic dye, indicating increased catabolic flux and cellular stress (Figure 3C, also refer to Figure S2 in Supplementary data). Treatment with the 12.5 mg/mL Trc mix alone led to 52% culture survival, correlating with the determined IC_50_ at 11.4 mg/mL against this *Candida* strain (Table 3 and Table S2 in Supplementary data). These cultures exhibited the most cellular stress, while 4/6 of the formulations with the 12.5 mg/mL Trc mix resulted in <50% culture survival (Table 2, Figure 3C). This strongly indicated that the cellulose-type formulants did enhance the anti-*Candida* activity of the Trc mix (Table 2, Figure 3). 

Cultures treated with the Trc mix formulated by cellulose derivatives with lower viscosity, such as KLUE and KLUL, showed higher survival than the control. This indicated that these cellulose derivatives did not enhance or stabilise the activity of the Trc mix at this concentration. The formulations of the Trc mix in A4M, E4M, E10M and K15M were selected for further investigation because they exhibited the same or better activity than the control preparation, specifically at the lower concentrations (Table 2, Figure 3B,C).

The survival within some of the treated cultures may be due to the presence of biofilm persister cells [50], as a mature biofilm cell population already exists in the 48 h *C. albicans* cultures, as determined in a parallel study by our group. *Candida* persister cells are defined as a subpopulation of cells in biofilms that survive antifungal drug treatment [50,51,52]. These persister cells tend to shift their metabolism to carbon storage and down-regulate glycolysis [50,52], and are therefore geared towards anabolism. This could curtail the availability of reducing agents from catabolism for the conversion of resazurin to resorufin in the antifungal assay, while stressed cells may increase the catabolic flux through glycolysis to cope with, for example, osmotic stress. We observed “negative” inhibition in many of the cultures, indicating a higher reduction of resazurin than in the control cultures (Figure 3, also refer to Figure S2 in Supplementary data). This indicated an increase in the availability of reducing agents, and therefore an increase in catabolism, which is the result of a stress response. The most stressed cell cultures were observed in the control Trc mix and at the lowest concentration of the 12.5 µg/mL Trc mix in the formulations, with 29–58% survival (Table 2, Figure 3, Figure S2 in Supplementary data). However, some cultures did not exhibit the stress response, which indicated that they may contain surviving cells (persister cells) of the early biofilm population present in the 25.5 h old cultures.

To follow the oligomerisation and conformational changes of the peptides in the Trc mix, we assessed the same formulations at 1 h and 20 h utilising fluorescence. The Trcs have three aromatic fluorophores, namely Phe, Tyr and Trp. We followed the fluorescence of Trp, as it has the largest resonance energy transfer, absorptivity and quantum yield [53], as well as being very sensitive to its environment in terms of peptide conformational charges and solvent changes [53,54,55]. After 4 h, about 20–24% of the Trp signal was lost for all the formulations and the control Trc mix. After 20 h, the control lost about 45% of its Trp signal, while the six formulations showed better stabilisation of the fluorescence signal over time (Tables S3–S5 in Supplementary data). The lower quantum yield of Trp could be due to either the exposure of the Trp residue to the polar solvent, polar groups in the celluloses, interaction with polar groups [32] (Lys^9^, Orn^9^, Gln^6^, Asn^5^ and Tyr^7^ side chains) and/or due to aromatic stacking [32,47]. Juhl et al. [46] showed that D-Phe^1^, residue 4 (D-Trp^4^ or D-Phe^4^) and Orn^9^ are most influenced by the interaction with glucose and its cellulose-type tetramer (cellotetraose), while Asn^5^ and Tyr^7^ are also influenced. A red-shifted emission maximum would be observed if the Trp residues were exposed to polar moieties in the formulants. However, the emission maximum of the formulations was comparable to that of Trc mix alone at 340 ± 1 nm. Part of the loss of the control Trc mix could therefore be explained as peptide self-assembly leading to self-quenching. The lower loss in the formulations indicates conformational changes that may involve changes in the aromatic stacking and participation of protruding polar side chains of the peptide with the formulants. The association with the plastic surface of the 96-well plate could also lead to the loss of Trp fluorescence. The association with various materials, including plastics and cellulose, was illustrated in a study by Van Rensburg [43,45]. However, the fact that the cellulose derivatives stabilised Trp fluorescence can be contributed to the interaction of cellulose derivatives with the cyclodecapeptides in the Trc mix. This interaction can prevent or slow down the formation and dynamic rearrangement of oligomers [40,56,57].

**Figure 4:**
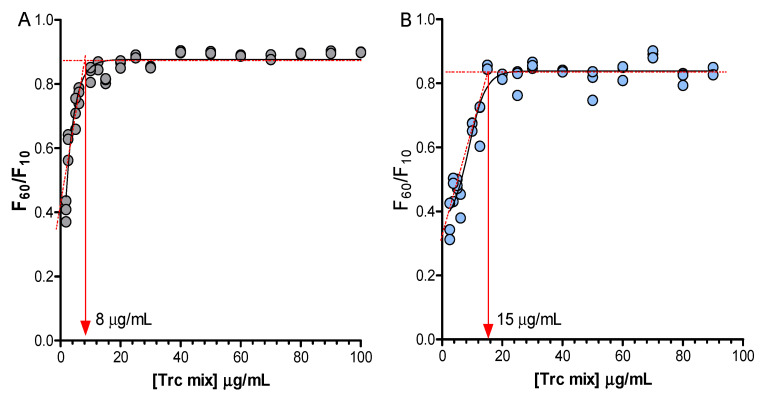
Monitoring the influence of the formulation on the oligomerisation concentration of the cyclodecapeptides in the Trc mix. Control (**A**); Trc mix: E4M (1:4) (**B**). Fluorescence excitation and emission of the Trc mix (1.25–100 µg/mL) were at 280 and 348 nm, respectively. Three repeats of each preparation are shown via scatter dot plots. Continual readings were taken for the first 60 min with F60/F10 (the reading taken at 60 min/reading taken at 10 min). A sigmoidal curve with a variable slope was fitted to each of the data sets (R2 > 0.99) and the intercept between the slope and the plateau was taken as the critical oligomerisation concentration.

The linear correlation between the Trc mix concentrations and the total fluorescence intensity is lost if the Trp residue participates in oligomerisation. The nonlinear change of Trp fluorescence at a specific concentration with the increase in the Trc mix concentration can therefore be used to determine the critical oligomerisation concentration. If the concentration of the Trc mix is high enough to result in the formation of large oligomers, it can lead to settling out of the solution and/or quenching of Trp fluorescence because of the aromatic stacking of aromatic amino acid residues (π-π interactions) [53,54,55]. The change in the environment of the peptide molecules can result in a change in hydrogen-bonded structures, weak noncovalent interaction and aromatic amino acid stacking, leading to a different critical oligomerisation concentration. Monitoring of Trp fluorescence over a broader concentration range of the Trc mix and the formulations revealed the critical concentration in which the Trp environment changes, related to the critical concentration of oligomerisation/aggregation. The change in the first 60 min was investigated by measuring the total Trp fluorescence over the Trc mix dose-response concentration range (100 to 1.25 µg/mL) with E4M and A4M as representative cellulose formulants. Our results corroborated previous observations [34,36,39,40,46] that the Trc mix environment has a major influence on oligomerisation behaviour (Figure 4). 

**Figure 5:**
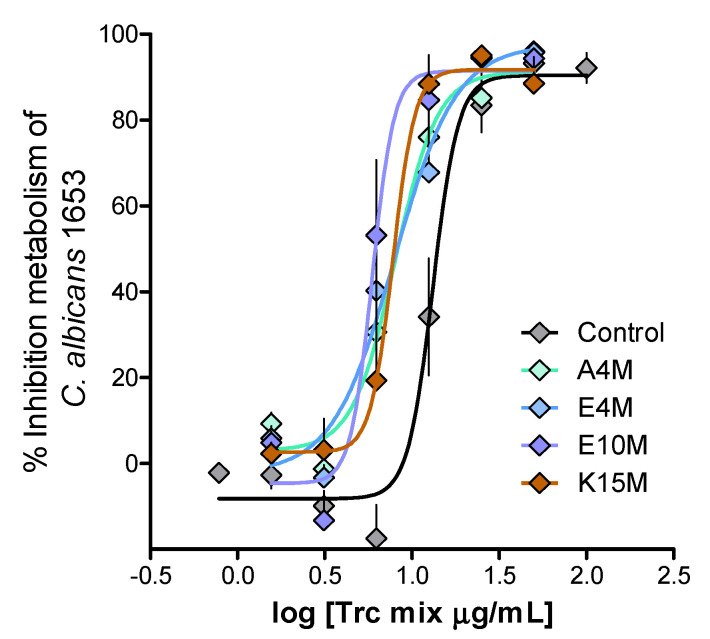
Representative dose-response curves of the Trc mix alone and the selected fresh 1:4 formulations. The average of four biological repeats (*n* = 16–30 dose-response curves) shows the activity of the Trc mix and its formulation against *C. albicans*. Each data point depicts the average and the standard error of the mean (SEM), and the curves were fitted with a sigmoidal curve with a variable slope.

From these studies, we determined the Trc mix critical oligomerisation concentration of the Trp-containing peptide as 8 µg/mL (~6 mM). The Trc mix formulation with A4M and E4M presented critical oligomerisation concentrations as 17 µg/mL (~13 mM) and 15 µg/mL (~12 mM), respectively (Figure 4, A4M results not shown). This increase in its critical oligomerisation concentration in the presence of the selected cellulose-type formulants could explain the improvement of activity at lower concentrations (refer to Figure 5), as more peptides in its active conformation may be available. 

KLUE and KLUL had a more similar Trp fluorescence stabilisation over time than A4M, E4M, E10M and K15M, indicating similar structures. However, the latter four exhibited similar or better activity than the control preparation, and thus were selected for further investigation (Table 2, Figure 3). In subsequent studies, we considered the formulations of the 50 g/mL Trc mix with 50 µg/mL, 100 µg/mL and 200 µg/mL of A4M, E4M, E10M and K15M to yield formulation ratios of 1:1, 1:2 and 1:4, respectively. The formulations were incubated for 1 h (assumed as fresh) and 20 h (matured formulation) at ambient temperature (22 ± 2 °C). To fully assess these formulations, full dose-response assays were performed against *C. albicans*, as the high formulant concentration may lead to the masking of groups in the peptide structure that are necessary for recognising the target cells.

The anti-*Candida* activity of the Trc mix and its formulations returned IC_50_ values in a narrow range of 7–10 mg/mL for the fresh preparations and 2–8 mg/mL for the 20-h-matured preparations. These values were below the critical oligomerisation concentrations of 8–16 mg/mL that were determined for the Trc mix and selected formulations. A comparative summary of the anti-*Candida* activity parameters is given in Table 3. Figure 5 shows the comparison of dose-response curves for the 1:4 formulations and the control Trc mix.

Statistical comparison of the activity of the fresh versus matured formulations indicated significant differences between the anti-*Candida* activity of the Trc mix and a substantial number of the formulations (Table 3, Tables S6 and S7 in Supplementary data). When all the datasets were compared using the one-way Anova with the Bonferroni correlation test, the control at 1 h had significantly lower activity (*p* < 0.001–0.01) compared to all the E10M and A4M formulations at 20 h. The 1:4 E10M formulation was also significantly more active (*p* < 0.001–0.01) than the control and other formulations at 1 h and 20 h, except for 1:1 E4M (Table 3, Tables S7 and S8 in Supplementary data). This indicated that the formulation type, formulant ratio and maturation time all have an influence on activity. When we considered the haemolytic activity of the different Trc mix formulations (Table 3), there was no clear distinction between the different formulations and the control. The haemolytic activity was similarly modulated with an increase in activity over time; however, there was a slight decrease in activity with the four-fold increase in formulant concentration. The selectivity index followed a similar trend, with an increase in selectivity over time.

**Figure 6:**
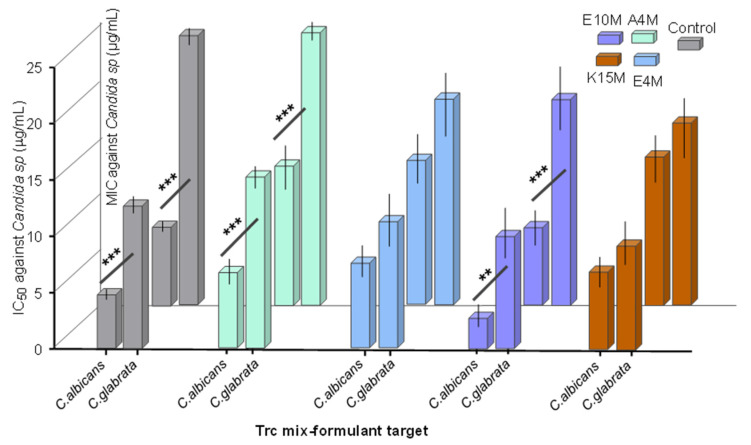
Comparison of the IC_50_ µg/mL against *C. albicans* and *C. glabrata* of 20-h-matured preparations (1:4 ratio). Data represent the mean of 3 biological repeats and 6–14 technical repeats with SEM. Unpaired Student’s t-test was done on each of the analysed groups, with ** *p* < 0.01 and *** *p* < 0.001.

**Figure 7:**
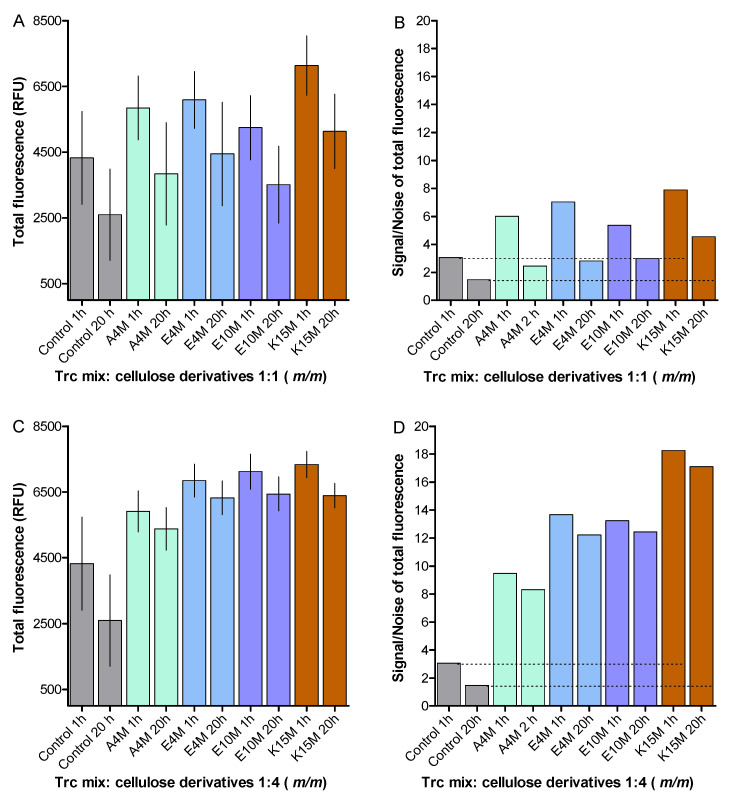
Comparison of the total fluorescence of the formulated Trc mix at 50 µg/mL and different ratios of cellulose derivatives at 1 h and after 20 h, showing the formulations with 1:1 (m/m) ratio (**A**), and those with 1:4 (m/m) ratio (**C**). Each bar represents the mean of 12 preparations and their determinations with SD. The S/N comparison of the respected datasets is shown in (**B**) and (**D**). The dotted lines show the comparison with the controls at 1 h and 20 h.

The activity was maintained if the IC_50_s were considered, but selectivity was moderately improved in all the fresh formulations (Table 3). Similar to the control, the 20-h-matured formulations showed improved activity and selectivity. Only one formulation, the 20-h-matured 1:4 E10M formulation, was significantly more active and selective than the control and other formulations (Table 3, Figure 6 and Figure 7A). However, if the whole dose response is considered for the 1:4 formulations versus that of the control Trc mix, there is a shift to the left for all the formulations, indicating an overall increase in activity, albeit it, a moderate increase (Figure 5).

The 20-h-matured 1:4 formulations of the Trc mix were also tested against another *Candida* species, namely *C. glabrata,* at the same cell density as *C. albicans*. The Trc mix and the Trc mix formulated with A4M and E10M had significantly lower IC_50_ values when targeting *C. albicans* than they did versus *C*. *glabrata,* while the K15M and E4M formulations exhibited similar activity (Figure 6). As these different target cells could have different growth rates and target concentrations; therefore, differences in MIC or IC_50_ values are to be expected. Interestingly, the fact that the activity of the K15M and E4M preparations of the Trc mix was similar against the two strains of *Candida* (Figure 6) could indicate that the formulation modulated the activity to a point that different growth profiles or target concentration had a minor influence.

To assess the stability of the 1:4 formulations over time, we compared the fluorescence of the fresh and matured formulations, containing the 50 µg/mL Trc mix with the 1:1 formulations (Figure 7). The change in total fluorescence of the formulated Trc mix at 1 h and 20 h of maturation is shown in Figure 7A,C. The 1:4 formulations yielded more stable Trp fluorescence over time with much less data scattering than the 1:1 formulations (Figure 7B,D). In the 1:4 formulations, the Trp fluorescence was found to be higher than that of the control, probably as the result of a change in the Trp environment and oligomerisation [40] (Figure 7A,C). This indicated that the 1:4 formulations were the more stable when the role of Trp was considered in tyrocidine oligomerisation/aggregation. Refer to Supplementary data, Tables S4 and S5, for the statistical analyses.

## 3. Materials and Methods

### 3.1. Materials

Commercial tyrothricin extract was supplied by Sigma-Aldrich (St Louis, MO, USA). Ethanol (EtOH), diethyl ether and acetone were supplied by Merck (Darmstadt, Germany). Analytical grade water (MQH_2_O) was prepared by filtering water from a reverse osmosis plant through a Millipore-Q^®^ water purification system (Milford, MA, USA). The 96-well black and transparent flat-bottom plates were aquired from Corning (Kennebunk, ME, USA). Benecel A4M (A4M), Benecel E4M (E4M), Benecel E10M (E10M), Benecel K15M (K15M), Klucel E IND (KLUE) and Klucel I IND (KLUI) were donated by Ashland, Covington, Kentucky, USA. Chitosan was purchased from Sigma-Aldrich (St Louis, MO, USA). Refer to Table 1 for more information on the formulations.

*Candida albicans* CAB1653, an environmental isolate from mosquito larvae from the Western Cape, South Africa, was donated from the CAB culture collection of Professor A. Botha in the Department of Microbiology, Stellenbosch University, South Africa; while *Candida glabrata* BG19, a clinical isolate, was donated by Dr. B. Bagheri, Tygerberg Medical School, Stellenbosch University, South Africa.

### 3.2. Preparation of the Culture Media

Sterile Petri dishes were obtained from Lasec (Ndabeni, South Africa) and Falcon tubes were supplied by Becton Dickson Labware (Lincoln Park, Chicago, IL, USA). Agar, sodium chloride, hydrochloric acid, tryptone and yeast extract were obtained from Merck (Darmstadt, Germany). RPMI 1640 medium was from Lonza (Walkersville, MD, USA) and resazurin sodium salt was from Sigma-Aldrich (St Louis, MO, USA). Mixed cellulose syringe filters (0.22 µm) were purchased from Merck-Millipore (Burlington, MA, USA).

### 3.3. Culturing and Growth Conditions of C. albicans and C. glabrata

Yeast cells from freezer stocks of *C. albicans* and *C. glabrata* were plated onto YPD agar plates (yeast extract 1%, peptone 2% *m*/*v* agar, glucose 2% *m*/*v* agar, 1.5% *m*/*v* agar) using sterile culturing techniques and incubated for 48 ± 2 h at 37 °C. This was followed by the inoculation of a single *Candida* colony in 20 mL YPD broth and growth for 17 h at 37 °C on a shaker at 150 rpm. RPMI media were then used to subculture the yeast at a final cell concentration of 5.5 × 10^5^ cells/mL.

### 3.4. Preparation of Formulations of Trc Mix

Tyrocidine mixture (Trc mix) was extracted from commercial tyrothricin using an optimised diethyl ether and acetone precipitation protocol [58]. The characterisation of the Trc mix can be found in Supplementary data (Figure S1, Table S1). Six different preparations of cellulose derivatives (Table 1) were made up to the stock concentrations of 1.00, 2.00 and 4.00 mg/mL in analytical quality water. The cellulose stock solutions were then sonicated for about 5 min to allow for complete solvation. These solutions were filter sterilised using a 0.2 µm sterile filter. The Trc mix was made up to the concentration of 1000 µg/mL in 15% (*v/v)* ethanol in MQH_2_O. The Trc mix and the formulants (cellulose derivatives) were mixed in varying ratios (1:1, 1:2, 1:4; m/m) and matured for 1 h (fresh) and 20 h (matured) at ambient temperature (22 ± 2 °C).

The formulations were mixed and incubated in a 96-well microtiter plate (dilution plate) and serially diluted to the desired concentration. A 10 µL aliquot of the Trc mix formulations were then transferred to another 96-well microtiter plate (assay plate) followed by the addition of 90 µL RPMI media containing the selected *Candida* cell culture.

### 3.5. Fluorescence Spectroscopy

To determine the biophysical characteristics of formulated Trc mix, formulants were prepared in 15% ethanol (*v*/*v*) followed by a 10-fold dilution by adding 90 µL of filter-sterilised MQH_2_O. The Trc mix formulations with the six cellulose-type formulants were composed as described above.

Each preparation was monitored individually, as technical repeats may not have identical behaviour because of the sensitivity of the Trc oligomerisation to plastic surface contact [43,44,45], time delay during pipetting, time delay after mixing, the sequence of adding components and small temperature variations. The formation of unstable oligomers and dynamic changes in oligomerisation can therefore be monitored and compared by assessing the scattering of data points. A scatter parameter that was defined as:

Average of 12 repeats/standard deviation = signal/noise = S/N.(1)

Readings at 340 nm of the 12 sample preparations at 12.5, 25 and 50 mg/mL were taken after 1 h and 20 h of maturation. For assays with the Trc mix ranging from 100 to 1.2 µg/mL, the fluorescence emission was measured at 340 nm over 60 min. For emission spectra, the fluorescence emission was scanned from 320 nm to 400 nm. For all the measurements, the excitation wavelength was 280 nm. The emission bandwidth was 20 nm with 10 flashes and a Z position of 2000 µm. All fluorescence readings were collected using the Tecan Spark 10M Multimode Microplate Reader.

### 3.6. Anti-Candida Assays

*C. albicans* CAB1653 cultures, exposed to the Trc mix and its formulations in 96-well microtiter plates, were incubated for 24 h at 37 °C and 90% humidity. This was followed by the addition of 10 μL resazurin dye solution (0.30 mg/mL) to each well and further incubation of 90 min for *C*. *albicans,* and 4 h for *C*. *glabrata* at 37 °C. The inhibition of metabolic activity was determined by measuring the fluorescence emission of the reduced metabolic dye resorufin using the Tecan Spark 10M Multimode Microplate Reader. Excitation and emission wavelengths were set at 560 nm and 590 nm (Em590), respectively. The measured fluorescence was converted to % inhibition of metabolism as described by the following equation:

(2) $$\;\%\;\mathrm{metabolic}\;\mathrm{inhibition}=100-\frac{100\times \left(\mathrm{Em}590\;\mathrm{of}\;\mathrm{culture}\;\mathrm{in}\;\mathrm{well}-\mathrm{mean}\;\mathrm{of}\;\mathrm{Em}590\;\mathrm{of}\;\mathrm{blank}\right)}{\left(\mathrm{mean}\;\mathrm{of}\;\mathrm{Em}590\;\mathrm{of}\;\mathrm{growth}\;\mathrm{metabolism}-\mathrm{mean}\;\mathrm{of}\;\mathrm{Em}590\;\mathrm{blank}\right)}\;$$

The negative control (0% metabolic inhibition) was cell cultures treated with 1.5% ethanol and the background (blank) was the RPMI medium containing no cells and only the solvent control.

Dose-response assays were done using 3 to 4 individual starter cultures, each divided into 8 to 20 subcultures. The method described by Rautenbach et al. [59] was utilised to calculate the minimum inhibition concentration (MIC) and IC_50_ (concentration that gives 50% metabolic inhibition) of the Trc mix and its formulations toward the two *Candida* species.

### 3.7. Haemolytic Activity

Packed erythrocytes, from an anonymous A + donor, were obtained from the Western Cape blood services in South Africa, adhering to the relevant ethics and legislation. The haemolysis assays were done according to Rautenbach et al. [22] with a few adaptations. Trc mix formulations of 1:1, 1:2 and 1:4 (*m*/*m*) with the different celluloses were individually prepared with doubling dilutions, starting at 500 µg/mL, in glass for each concentration. Following a 1 h and 24 h incubation, 50.0 µL was dried in a 96-well plate in a 60 °C oven overnight.

Washed erythrocytes in phosphate buffer saline (PBS) at 2% haematocrit (100 µL) were then added to each well and incubated at 37 °C for 2 h. The plates were then centrifuged (900× *g* for 10 min) and 10 µL of each well’s supernatant was transferred to a 96-well plate containing 90 µL PBS. The released haemoglobin of the lysed erythrocytes was measured at 415 nm using a Tecan Spark 10M Multimode Microplate Reader and controlled by the Spark Control^TM^ software, both provided by Tecan Group Ltd. (Mennedorf, Switzerland). The haemolytic activity was calculated using the following equation:

(3) $$\%\;\mathrm{haemolytic}\;\mathrm{activity}\;=100\times \frac{\mathrm{Abs}\;\mathrm{at}\;415\;\mathrm{nm}\;\mathrm{of}\;\mathrm{sample}\;-\mathrm{mean}\;\mathrm{Abs}\;\mathrm{at}\;415\;\mathrm{nm}\;\mathrm{of}\;\mathrm{background}}{\mathrm{mean}\;\mathrm{Abs}\;\mathrm{at}\;415\;\mathrm{nm}\;\mathrm{of}\;\mathrm{fully}\;\mathrm{lysed}-\mathrm{mean}\;\mathrm{Abs}\;\mathrm{at}\;415\;\mathrm{nm}\;\mathrm{of}\;\mathrm{background}\;}$$

The full lysis was determined with 10 µg gramicidin S, translating to 100 µg/mL in the assay in the lysis control wells. The negative control (background, 0% lysis) were erythrocytes in PBS.

## 4. Conclusions

In this study, we found that the formulation of the Trc mix with more viscose cellulose derivatives and a longer maturation of the formulation components resulted in the maintenance of, or a moderately improved activity against, *C. albicans* cell cultures. It was also found that the prolonged maturation of formulations enhanced the biological activity and selectivity of the Trc mix and its formulations. The A4M, E4M and K15M formulations maintained the activity and selectivity of the control Trc mix. The Trc mix formulated with E10M at 1:4 (m/m) had significantly enhanced activity and selectivity when targeting *C. albicans.* This indicated that the interaction of the Trc mix with the more viscous E10M and the longer maturation of the Trc formulation causes a conformational change and/or a change in the oligomeric distribution. The rearrangement of oligomers in the formulation possibly form smaller oligomers from which the proposed active dimers can be readily released [33].

The stability of the formulations and maintenance of anti-*Candida* activity and the improved, albeit with low selectivity, activity of the Trc mix peptides, would only allow for topical applications, such as oral and vaginal candidiasis. Tyrocidine-containing medicaments have been used in topical applications during the last 70 years, but they were more focused on bacterial infections [25,60,61]. It is therefore feasible to utilise a 1:4 formulation of a Trc mix with E10M in a topical formulation directed against *Candida* strains.

## Figures and Tables

**Table 1:** Summary of commercial, modified cellulose derivatives utilised as formulants for the Trc mix in this study.

Saccharide Derivative	Commercial ID Abbreviation	Modification	Viscosity Cps	Colour Code *
Methyl-cellulose	A4M	-CH_3_	3500–5600	
Hydroxy-propyl-methyl-cellulose	E4M	-CH_2_OH/ -CH_2_CH(OH)CH_3_	2700–5040	
Hydroxy-propyl-methyl- cellulose	E10M	-CH_2_OH/ -CH_2_CH(OH) CH_3_	7500–14,000	
Hydroxy-propyl-cellulose	KLUE	-CH_2_OH	200–600	
Hydroxy-propyl- cellulose	KLUL	-CH_2_OH	75–150	
Hydroxy-propyl-methyl-cellulose	K15M	-CH_2_OH/ -CH_2_CH(OH)CH_3_	10,000–18,000	

* The colour code was used in all graphs to ease the comparative interpretation.

**Table 2:** Survival (%) of 24 cultures after exposure to the Trc mix and its formulations with a variety of saccharides at the three selected concentrations.

	Trc Mix Concentration in Formulation		
Peptide Formulant	50 µg/mL	25 µg/mL	12.5 µg/mL
Control	4 *	16 *	52 *
A4M	0	0	29
E4M	0	0	37
E10M	0	4	42
KLUE	0	0	58
KLUL	0	8	54
K15M	0	4	37

* 55–60 cultures were treated for the control Trc mix.

**Table 3:** Summary of the IC_50_ values of different preparations of the Trc mix against *C. albicans*. Refer to Tables S6 and S7 for statistical analyses.

		IC_50_ ± SEM (µg/mL)		HC_50_ ± SEM (µg/mL)		Selectivity HC_50_/IC_50_	
Formulant or Drug	Trc Mix: Formulant (*m*/*m*)	Fresh 1 h, *n* = 16	Matured 20 h, *n* = 11–16	Fresh 1 h, *n* = 3	Matured 20 h, *n* = 3	Fresh, 1 h	Matured 20 h
Control	1:0	11.4 ± 1.3 *	5.3 ± 0.4	14 ± 0.7 ^#^	12 ± 0.4 ^#^	1.2	2.2
A4M	1:1	8.6 ± 1.7	4.6 ± 0.0	13 ± 0.2	10 ± 0.2	1.5	2.2
E4M	1:1	6.6 ± 1.2	5.4 ± 0.5	13 ± 0.8	10 ± 0.2	2.0	1.9
E10M	1:1	8.1 ± 1.3	5.7 ± 0.6	12 ± 0.3	10 ± 0.2	1.4	1.7
K15M	1:1	8.9 ± 1.1	7.5 ± 1.3	13 ± 0.4	12 ± 0.2	1.5	1.6
A4M	1:2	8.9 ± 0.6	4.9 ± 0.2	14 ± 0.5	11 ± 0.7	1.6	1.7
E4M	1:2	10 ± 0.8	6.8 ± 1.2	16 ± 1.5	12 ± 0.5	1.5	2.2
E10M	1:2	9.2 ± 0.3	6.2 ± 0.5	15 ± 0.5	14 ± 0.6	1.7	2.2
K15M	1:2	8.9 ± 0.9	5.1 ± 0.2	13 ± 1.1	11 ± 0.3	1.4	2.2
A4M	1:4	9.6 ± 1.3	5.6 ± 0.5	17 ± 2.0	13 ± 0.5	1.7	2.4
E4M	1:4	8.8 ± 1.4	6.5 ± 0.9	16 ± 0.6	14 ± 0.5	1.8	2.1
E10M	1:4	7.0 ± 0.8	2.3 ± 0.7	14 ± 0.1	12 ± 0.3	1.9	5.3
K15M	1:4	8.2 ± 1.3	6.5 ± 1.2	14 ± 0.9	13 ± 0.3	1.7	2.0

* Average of 30 dose responses; ^#^ Average of 8 dose responses.

## Data Availability

Raw data are available from the corresponding authors.

## Supplementary Materials

The following are available online at https://www.mdpi.com/article/10.3390/antibiotics10050597/s1, Figure S1: UPLC chromatograms of commercial tyrothricin complex with the untreated tyrothricin and the precipitate of the DEE:acetone wash containing the Trc mix, Table S1: Percentage abundance and theoretical mass (mg) yield of the major Trc analogues present within the commercial tyrothricin complex before and after the wash with DEE:acetone, Table S2: Comparison of IC_50_ and MIC values of selected antifungal compounds/drugs against planktonic cultures of *C. albicans* CAB1653. Figure S2: Correlation between metabolic inhibition of a range of C. albicans cell concentrations versus Trc mix concentrations, Table S3 Summary of % relative fluorescence unit loss measured for each of the eight formulants of Trc mix 1:1 (*m*/*m*) from 1 to 4 h and 1 to 20 h of maturation. Table S4: Statistical comparison of Trp fluorescence between different Trc mix of fresh (1 h) and matured (20 h) preparations, Table S5: One-way Anova statistical comparison Trp fluorescence between different formulations of Trc mix at 20 hours of maturation. Table S6: Student t-test statistical comparison between different Trc mix of fresh (1 h) and matured (20 h) formulations of the observed inhibition parameters against planktonic cultures of *C. albicans* CAB1653. Table S7: One way Anova statistical comparison of IC_50_ (µg/mL) value correlation between the different preparations of Trc mix (1h vs 20h), Table S8: One-way Anova statistical comparison of IC_50_ (µg/mL) between different preparations of Trc mix after 20 h of maturation.
