# Supplementary material for: The Influence of Cellulose-Type Formulants on Anti-Candida Activity of the Tyrocidines
Source: Antibiotics (Basel). 2021 May 18;10(5):597. doi: 10.3390/antibiotics10050597 (PMC8157355; doi:10.3390/antibiotics10050597)
Supplement: Supplementary file 1 [file antibiotics-10-00597-s001.zip › antibiotics-1168811-supplementary.pdf]

antibiotics

SUPPLEMENTARY DATA

The Influence of Cellulose-Type Formulants on Anti-*Candida* Activity of the Tyrocidines

Yasamin Masoudi, Wilma van Rensburg *, Bernice Barnard-Jenkins and Marina Rautenbach *

BIOPEP™ Peptide Group, Department Biochemistry, Stellenbosch University, Private Bag X1, Matieland, 7602 Stellenbosch, South Africa; yasamin@sun.ac.za (Y.M.); bbarnard@sun.ac.za (B.B.-J.)
* Correspondence: wvr@sun.ac.za (W.v.R.); mra@sun.ac.za (M.R.); Tel.: +27-21-808-5872/8 (M.R.)

Peptide purification and characterization

Tyrocidine mixture (Trc mix) was extracted from commercial tyrothricin using an optimised diethyl ether (DEE) and acetone precipitation protocol [42] and subjected to RP-HPLC (Figure S1). The insolubility of tyrocidine analogues (Trcs) and solubility of the hydrophobic linear gramicidins (Grms) in DEE-acetone, due to their different hydrophobic properties, allowed for the separation of the two groups of peptides from the tyrothrcin mixture. However, is has been observed that the DEE-acetone wash results in the loss of some Trcs analogues. Therefore, each complex (tyrothricin and the Trcs fraction) were subjected to UPLC-ESMS to compare the abundance of different peptides analogues present within the preparation (Figure S1). The UPLC-ESMS was performed based on an optimised method [42].

The DEE-acetone wash resulted in complete loss of minor analogues and significant loss of some of the major analogues present within the fraction despite the overall increase in purity with the removal of the Grms (Table S2).

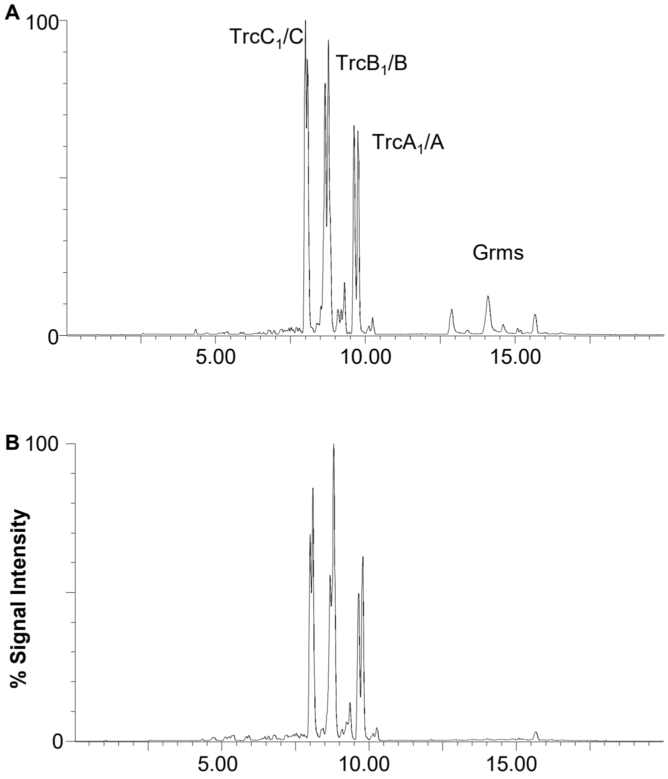

Figure S1: UPLC chromatograms of commercial tyrothricin complex with (**a**) the untreated tyrothricin, (**b**) the precipitate of the DEE: acetone wash containing the Trc mix.

Table S1: Percentage abundance and theoretical mass (mg) yield of the major Trc analogues present within the commercial tyrothricin complex before and after the wash with DEE: Acetone.

	**%Abundance** [a]		**Theoretical mass (mg) yield of the major analogue** [c]		**%abundance Trc mix**	
	Tyrothricin complex	Extracted Trc mix	Tyrothricin complex	Extracted Trc mix	Vosloo [20]	Troskie *et al.* [6]
$TrcC_1$	14.2	12.4	2.13	1.27	1.6	12.5
TrcC	13.6	15.0	2.04	1.53	10.7	14.8
$TpcC_1$	1.1	1.2	0.17	0.12	0.3	-
TpcC	1.5	2.9	0.23	0.3	2.5	1.7
$TrcB_1$	14.4	9.3	2.16	0.95	3.5	19.2
TrcB	15.2	22.9	2.28	2.34	9.4	18.2
TrcB′	3.9	-	0.59	-	8.5	4.0
TpcB	2.7	3.8	0.41	0.27	6.2	1.0
$TrcA_1$	9.9	10.4	1.49	1.06	4.4	15.8
TrcA	9.6	13.0	1.44	1.32	21.1	12.9
TpcA	0.7	1.4	0.11	0.1	6.3	Trace
IGB	2.2	-	0.33	-	0.6	-
VGA	4.1	-	0.62	-	6.1	-
VGB	1.8	-	0.27	-	0.6	-
%Trcs [b]	86.8%	92.1%	13.05 mg	9.26 mg	81.8%	100.1%

[a] % Abundance was calculated by expressing the peak area of each peptide as a percentage of the sum of the peak areas of all peptides present in the extract. It was assumed that the response factors of all peptides are similar due to their analogue structure.
[b] % Trcs was determined by the sum of the peak areas of all the Trcs present in the tyrothricin complex and each of the extracts.
[c] The mass (mg) of the predominant analogues present in the tyrothricin complex and each of the extracts was calculated by multiplying of % abundance by the total mass (mg) of in the tyrothricin complex and each of the extracts.

Activity of Trc mix and commercial antifungal compounds against *Candida albicans*

Table S2: Comparison of IC_{50} and MIC values of selected antifungal compounds against planktonic cultures of *C. albicans* CAB1653. Tabulated IC_{50} and MIC values represents the mean of 3–4 biological repeats and 12–32 technical repeats with SEM.

Drug or peptide	IC_{50} (μM)	MIC (μM)
Trc mix	11 ± 1.3	12.5–25
Caspofungin	4.8 ± 0.7	8.6 ± 1.1
Amphotericin B	13 ± 2.9	23 ± 4.2
Fluconazole	>325	>325

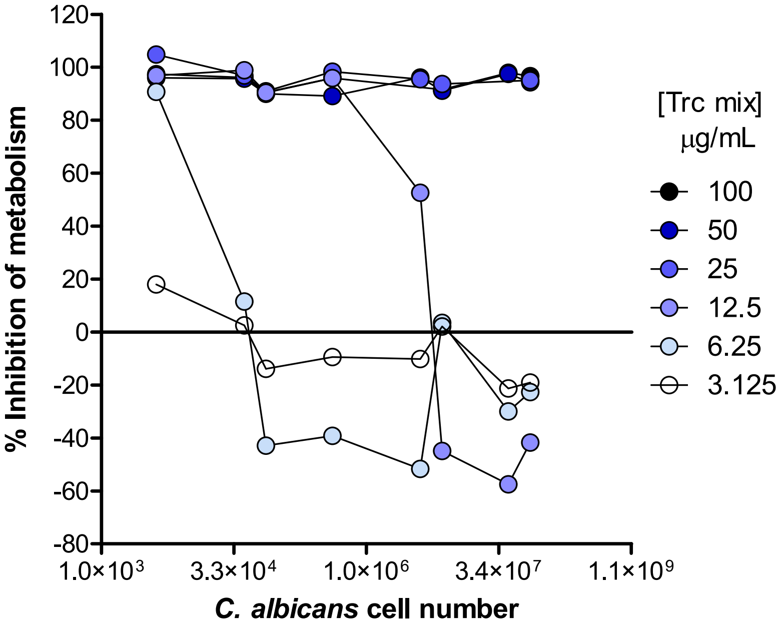

Figure S2: Correlation between metabolic inhibition of a range of *C. albicans* cell concentrations versus Trc mix concentrations. The values below 0% inhibition indicate higher conversion of the metabolic dye versus the control cultures. Each data point is the average determined for two cultures.

Fluorescence analysis of Trc mix and its formulations

Table S3: Summary of % relative fluorescence unit (RFU) loss measured for each of the eight formulants of Trc mix 1:1 (*m*/*m*) from 1 to 4 h and 1 to 20 h of maturation. The relative loss is also indicated as a heat map with blue the lowest lost and red the highest loss.

	**Mean of % RFU loss from 1 hour**	
**Cellulose derivatives**	**4 hours of maturation**	**20 hours of maturation**
Control	23	45
A4M	22	32
E4M	20	25
E10M	24	31
KLUE	19	26
KLUL	22	30
K15M	20	27

Table S4: Student t-test statistical comparison of Trp fluorescence between different Trc mix of fresh (1 hour) and matured (20-hour) preparations. Tabulated Trp fluorescence represents the mean 12 preparations with SD. Unpaired Student t-test was done on each of the analysed pairs.

Formulant	Fresh sample (1 hour) mean ± SD	Matured sample (20 hours) mean ± SD	P-value	% Change 1 h to 20 h
Trc mix: cellulose derivatives 1:1 (*m*/*m*)				
A4M	5844 ± 72.0	3839 ± 1557	0.0010	34
E4M	6087 ± 249.7	4443 ± 1573	0.0044	27
E10M	5244 ± 282.5	3511 ± 1173	0.0007	33
K15M	7130 ± 260.7	5133 ± 1129	<0.0001	28
Trc mix: cellulose derivatives 1:4 (*m*/*m*)				
A4M	5910 ± 623.3	5381 ± 646.8	ns	9
E4M	6850 ± 501.1	6326 ± 516.9	ns	8
E10M	7120 ± 537.2	6443 ± 518.0	ns	10
K15M	7335 ± 401.6	6392 ± 373.6	ns	13
Control	5539 ± 4481	2345 ± 1584	0.0277	58

Table S5: One-way Anova statistical comparison Trp fluorescence between different formulations of Trc mix at 20 hours of maturation. Analysed Trp fluorescence represents the mean of 12 preparations with SD (refer to Table S3). One-way Anova with Bonferroni correlation test was done between the selected data sets.

			20 hours maturation								
			1:4				1:1				
			K15M	E10M	E4M	A4M	K15M	E10M	E4M	A4M	Control
20 hours of maturation	1:4	K15M		ns	ns	ns	ns	0.001	ns	<0.01	0.001
		E10M			ns	ns	ns	0.001	ns	0.001	0.001
		E4M				ns	ns	0.001	<0.05	<0.01	0.001
		A4M					ns	ns	0.001	ns	0.001
	1:1	K15M						ns	ns	ns	0.001
		E10M							ns	ns	ns
		E4M								ns	<0.05
		A4M									ns

Statistical analysis of activity data

Table S6: Student t-test statistical comparison between different Trc mix of fresh (1 h) and matured (20 h) formulations of the observed inhibition parameters against planktonic cultures of *C. albicans* CAB1653. Tabulated IC_{50} and MIC values (μg/mL) represents the mean of 3–4 biological repeats and 12–30 technical repeats with SEM. Unpaired Student t-test was done on each of the analysed pairs and only those with significant differences are shown.

Formulation at 1-hour vs 20-hours	IC_{50} ± SEM (μg/mL) 1 hour	IC_{50}± SEM (μg/mL) 20 hours	P value
Control	11.4 ± 1.3	5.3 ± 0.4	0.0003
Trc mix:A4M (1:2)	8.9 ± 0.6	4.9 ± 0.2	<0.0001
Trc mix:A4M (1:4)	9.6 ± 1.3	5.6 ± 0.5	0.0196
Trc mix:E4M (1:2)	10.3 ± 0.8	6.8 ± 1.2	0.0143
Trc mix:E10M (1:2)	9.2 ± 0.3	6.2 ± 0.5	<0.0001
Trc mix:E10M (1:4)	7.0 ± 0.8	2.3 ± 0.7	0.0002
Trc mix:K15M (1:2)	8.9 ± 0.9	5.1 ± 0.3	0.0017
**Formulation at 1-hour vs 20-hours**	**MIC ± SEM (μg/mL) 1 hour**	**MIC± SEM (μg/mL) 20 hours**	**P value**
Control	14.9 ± 1.5	7.5 ± 0.5	0.007
Trc mix:A4M (1:1)	14.0 ± 2.4	6.7 ± 0.5	0.0203
Trc mix:A4M (1:4)	14.8 ± 1.6	8.8 ± 0.9	0.0065
Trc mix:E10M (1:4)	10.1 ± 1.2	4.6 ± 0.8	0.0023

Table S7: One way Anova statistical comparison of IC50 (μg/mL) value correlation between the different preparations of Trc mix (1 h vs 20 h). The analysed IC_{50} values were the mean of 3–4 biological repeats and 12–30 technical repeats with SEM. One-way Anova with Bonferroni correlation test was done between each of the selected data sets.

			20 hours of maturation												
			1:4				1:2				1:1				
			K15M	E10M	E4M	A4M	K15M	E10M	E4M	A4M	K15M	E10M	E4M	A4M	Control
1 hour of maturation	1:4	K15M	ns	<0.01	ns	ns	ns	ns	ns	ns	ns	ns	ns	ns	ns
		E10M	ns	<0.001	ns	ns	ns	ns	ns	ns	ns	ns	ns	ns	ns
		E4M	ns	<0.001	ns	ns	ns	ns	ns	ns	ns	ns	ns	ns	ns
		A4M	ns	<0.001	ns	ns	ns	ns	ns	<0.05	ns	ns	ns	<0.05	ns
	1:2	K15M	ns	<0.001	ns	ns	ns	ns	ns	ns	ns	ns	ns	ns	ns
		E10M	ns	<0.001	ns	ns	ns	ns	ns	ns	ns	ns	ns	ns	ns
		E4M	ns	<0.001	ns	ns	ns	ns	ns	<0.05	ns	<0.05	<0.05	<0.05	<0.05
		A4M	ns	<0.001	ns	ns	ns	ns	ns	ns	ns	ns	ns	ns	ns
	1:1	K15M	ns	<0.001	ns	ns	ns	ns	ns	ns	ns	<0.05	ns	ns	ns
		E10M	ns	<0.01	ns	ns	ns	ns	ns	ns	ns	ns	ns	ns	ns
		E4M	ns	<0.01	ns	ns	ns	ns	ns	ns	ns	ns	ns	ns	ns
		A4M	ns	<0.001	ns	ns	ns	ns	ns	ns	ns	ns	ns	ns	ns
		Control	ns	<0.001	ns	<0.01	<0.01	<0.001	ns	<0.01	ns	<0.01	<0.01	<0.001	<0.001

Table S8: One-way Anova statistical comparison of IC_{50} (µg/mL) between different preparations of Trc mix after 20 hours of maturation. The analysed IC_{50} values were the mean of 3–4 biological repeats and 12–30 technical repeats with SEM. One-way Anova with Bonferroni correlation test was done between each of the selected data sets.

			20 hours of maturation												
			1:4				1:2				1:1				
			K15M	E10M	E4M	A4M	K15M	E10M	E4M	A4M	K15M	E10M	E4M	A4M	Control
20 hour of maturation	1:4	K15M		<0.01	ns	ns	ns	ns	ns	ns	ns	ns	ns	ns	ns
		E10M			<0.01	<0.001	<0.001	<0.001	<0.01	<0.01	<0.001	<0.001	ns	<0.05	ns
		E4M				ns	ns	ns	ns	ns	ns	ns	ns	ns	ns
		A4M					ns	ns	ns	ns	ns	ns	ns	ns	ns
	1:2	K15M						ns	ns	ns	ns	ns	ns	ns	ns
		E10M							ns	ns	ns	ns	ns	ns	ns
		E4M								ns	ns	ns	ns	ns	ns
		A4M									ns	ns	ns	ns	ns
	1:1	K15M										ns	ns	ns	ns
		E10M											ns	ns	ns
		E4M												ns	ns
		A4M													ns
